# Toxin-Antitoxin Systems of *Staphylococcus aureus*

**DOI:** 10.3390/toxins8050140

**Published:** 2016-05-05

**Authors:** Christopher F. Schuster, Ralph Bertram

**Affiliations:** 1 Section of Microbiology & MRC Centre for Molecular Bacteriology and Infection, Imperial College London, London SW7 2AZ, UK; c.schuster@imperial.ac.uk; 2 Interfakultäres Institut für Mikrobiologie und Infektionsmedizin, Lehrbereich Mikrobielle Genetik, University of Tübingen, 72076 Tübingen, Germany; 3 Klinikum Nürnberg Medical School GmbH, Research Department, Paracelsus Medical University, 90419 Nuremberg, Germany

**Keywords:** *Staphylococcus aureus*, toxin-antitoxin system, plasmid addiction, MazEF, YefM-YoeB, RNase, SprA1-SprA1_AS_, SprFG, TenpIN, Omega-Epsilon-Zeta

## Abstract

Toxin-antitoxin (TA) systems are small genetic elements found in the majority of prokaryotes. They encode toxin proteins that interfere with vital cellular functions and are counteracted by antitoxins. Dependent on the chemical nature of the antitoxins (protein or RNA) and how they control the activity of the toxin, TA systems are currently divided into six different types. Genes comprising the TA types I, II and III have been identified in *Staphylococcus aureus*. MazF, the toxin of the *mazEF* locus is a sequence-specific RNase that cleaves a number of transcripts, including those encoding pathogenicity factors. Two *yefM-yoeB* paralogs represent two independent, but auto-regulated TA systems that give rise to ribosome-dependent RNases. In addition, *omega*/*epsilon*/*zeta* constitutes a tripartite TA system that supposedly plays a role in the stabilization of resistance factors. The SprA1/SprA1_AS_ and SprF1/SprG1 systems are post-transcriptionally regulated by RNA antitoxins and encode small membrane damaging proteins. TA systems controlled by interaction between toxin protein and antitoxin RNA have been identified in *S. aureus* *in silico*, but not yet experimentally proven. A closer inspection of possible links between TA systems and *S. aureus* pathophysiology will reveal, if these genetic loci may represent druggable targets. The modification of a staphylococcal TA toxin to a cyclopeptide antibiotic highlights the potential of TA systems as rather untapped sources of drug discovery.

## 1. Introduction

*Staphylococcus aureus* is a ubiquitous Gram positive commensal, colonizing about one third of the world’s human population. Usually asymptomatic, it is also a notorious etiologic agent of a multitude of diseases. These range from superficial infections to life-threatening inflammation and sepsis and are frequently caused by multi-drug resistant strains [1,2]. *S. aureus* deconvolves its pathogenicity via numerous virulence factors, including secreted toxins [3]. Commonly, bacterial toxins are considered to be poisonous compounds associated with pathogens targeting other bacteria or host cells. Bacterial toxins vary markedly in structure and function, depending in part on whether they are produced by invasive- or non-invasive pathogens [4].

Notably, however, the term “bacterial toxin” extends beyond factors that primarily harm other organisms, but also includes proteins which interfere with vital cellular functions of the prokaryotic cell from which they are encoded. By default, such toxins are negatively controlled by other proteins or RNAs to form so called toxin-antitoxin (TA) systems [5,6,7]. TA systems are present also in *S. aureus*, but were originally identified through their role in plasmid maintenance in other bacteria. A brief history of this field of research and functions of TA systems is given in the following. The episomally located *ccdBA* locus encodes the gyrase inhibitor CcdB (the toxin portion of the TA system), which is held in check by the antitoxin protein CcdA. CcdA is instable and if it cannot be replenished (e.g., after plasmid loss), cells are eradicated by the action of unleashed CcdB [8,9]. Similarly, faithful partitioning of plasmids is ensured by the *hok*-*sok* TA system identified on the R1 plasmid of Enterobacteriaceae. In this case, the membrane-targeting Hok toxin is translationally controlled by the small non-coding RNA antitoxin *sok* [10].

The plasmid addiction modules *ccdBA* and *hok*-*sok* are paradigms of type II or type I TA systems, respectively. The activity of type I TA toxins, most of which are small hydrophobic proteins corrupting cell envelopes, is controlled by an RNA antitoxin that inhibits translation and/or promotes degradation of toxin mRNA. Cognate toxin and antitoxin loci of type I TA systems are usually overlapping [11,12,13]. By contrast, in type II TA systems, both toxin and antitoxin are proteins, whose genes are canonically positioned adjacently within a bi-cistronic operon. Type II TA toxins are generally larger than those of type I TA systems with modes of action including cleavage of nucleic acids [14], inhibition of DNA replication [15], inhibition of ribosome assembly [16] and interference with peptidoglycan synthesis [17]. To date, a total of six different types of TA systems has been proposed, three of which are characterized by only one verified example each [18,19,20]. Like in type II systems, the antitoxins of TA types IV, V and VI are proteins, whereas in type III TA systems, a toxin protein is controlled by direct interaction with an antitoxin RNA [21]. TA systems are frequently auto-regulated and activated through alterations in the ratio between antitoxin-bound and free toxin protein. This can be achieved by a decrease in antitoxin RNA abundance or by proteolytic degradation of protein antitoxins, triggered by environmental changes or different kinds of stresses. A number of reviews provide a comprehensive picture of TA systems in general [5,6,7,22,23] and those regulated by RNAs in particular [11,12].

Sophisticated *in silico* methods and algorithms have identified TA systems in numerous prokaryotes [21,24,25,26], with abundances ranging from nil to several dozen per genome [22]. In contrast to the systems initially detected on plasmids, many more were found to be encoded chromosomally with their functions extending beyond plasmid maintenance systems. Instead, TA systems are most frequently reported in the context of bacterial stress response, programmed cell arrest or cell death, the formation of drug-tolerant persister cells, pathogenicity, biofilm formation and phage defense [23,27,28,29,30,31,32,33,34]. Among the staphylococci, TA systems have so far been characterized in the non-pathogenic *S. equorum* [35,36] and in *S. aureus*. For the latter, we here provide an overview on both experimentally verified and *in silico* predicted systems, all of which belong to TA types I, II and III.

## 2. Type I TA Systems

Research on type I TA systems in *S. aureus* was strongly promoted by investigations of the enterococcal plasmid pAD1 [37,38], and by the identification of numerous small non-coding RNAs [39,40,41,42]. Among a number of postulated TA loci in the *S. aureus* genome, only two type I TA systems of this organism have been experimentally characterized to date.

### 2.1. SprA1/SprA1_AS_ and Further Fst-Like Systems

**Figure 1:**
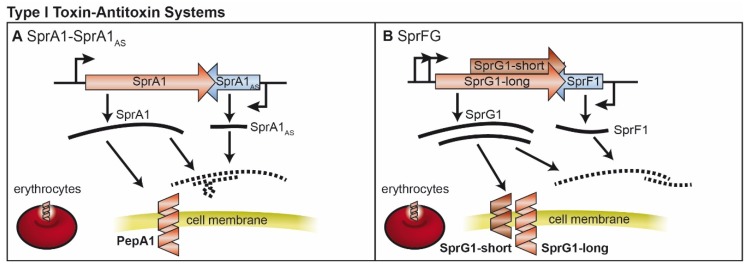
Type I toxin-antitoxin systems in *S. aureus* (**A**) The SprA1-SprA1_AS_ toxin-antitoxin system. Toxin (SprA1) and antitoxin (SprA1_AS_) RNAs are transcribed from convergent promoters. The toxin RNA (SprA1) gives rise to a cytotoxic peptide PepA1 that is able to disrupt the host membrane and erythrocytes. The antitoxin RNA is able to inhibit toxin synthesis by interactions with the non-overlapping areas; (**B**) The SprFG1 toxin-antitoxin system. Similar to (**A**) but from one toxin RNA two peptides of different lengths (SprG1-short, SprG1-long) are produced. In addition, the toxin-antitoxin RNA interaction occurs via the overlapping region. Not drawn to scale.

The TA system *par* of the *Enterococcus faecalis* pAD1 vector was the first plasmid maintenance, or post-segregational killing system to be identified in a Gram positive bacterium [37]. The *par* locus encodes two short and convergently transcribed RNAs, designated RNA I and RNA II, the first of which encodes the peptide Fst which is 33 amino acids in size. The sequence of the shorter RNA II is largely complementary to RNA I, rendering it capable of inhibiting Fst translation. Weaver and colleagues demonstrated that Fst of pAD1 affected growth and cell morphology of *S. aureus* when inducibly expressed [43]. The authors also identified an Fst-RNA II locus on *S. aureus* plasmid VRSAp, which was soon followed by the revelation of further Fst-like TA systems in more than a dozen *S. aureus* strains and plasmids [24,44,45]. These systems were found to be episomally or chromosomally encoded and associated with theta-replicating plasmids and the staphylococcal pathogenicity island SaPIbov2. In the course of a whole transcriptome analysis in the N315 strain [39], two of the small RNAs detected were later verified as antitoxins of type I TA systems (Figure 1A). One of the RNAs initially designated Teg152 resembles RNA II of the Fst-system. It was later renamed to SprA1_AS_ and is encoded together with the toxin gene *sprA1* in the νΣαβ pathogenicity island of strains including N315, Newman, NCTC8325 and USA300. As analyzed by the Felden group, expression of both genes was constitutive with a molar excess of SprA1_AS_ of about 35-90-fold relative to SprA [46]. Both RNAs fold into structures containing intramolecular double-stranded regions and interact via a specific duplex formed by non-overlapping 5′ domains of each molecule. Toeprinting analysis using purified *S. aureus* ribosomes verified translational repression of SprA1 by SprA1_AS_ presumably via prevention of ribosome loading. An SprA1 encoded peptide termed PepA1 inhibited *S. aureus* growth *in vivo* and this effect could be attenuated upon expression of SprA1_AS_ in trans [46]. In a subsequent study, PepA1 was characterized as a 30 amino acid helical peptide capable of corrupting the *S. aureus* membrane [47]. Resulting from decreased SprA1_AS_ levels, PepA1 expression is favored in an acidic environment and during oxidative stress. It has been postulated that the activation of PepA1 by reactive oxygen species represents a survival strategy of *S. aureus* after internalization into the phagolysosome of host immune cells. According to this theory, PepA1 will kill most of the rapidly dividing internalized bacteria, to allow a minor fraction of slowly diving bacteria to persist. The concomitant damage of phagolysosomal membranes by PepA1 furthermore favors bacterial escape [47]. As hypothesized, PepA1 may also play a role in the modulation of iron acquisition by lysis of erythrocytes. Although the actual function(s) of PepA1 require revelation, SprA1-SprA1_AS_ is currently the most extensively characterized type I TA system of *S. aureus*.

### 2.2. SprF/SprG

The *txpA-ratA* family represents another kind of type I TA systems [24], with the TxpA toxin counteracted by the *ratA* antitoxin. The first TxpA representative was identified in the chromosome of *Bacillus subtilis* [48] and an *in silico* analysis identified two possible TxpA homologs in *S. aureus* [24] (Figure 1B). Of these, SprG1, encoded on the mobile genetic element ΦSa3 PI of strain N315, was experimentally characterized [49]. The 3′ end of the *sprG1* toxin gene is overlapped by the convergently transcribed SprF1 non-coding RNA (Figure 1B). Notably, SprG1 can be either produced from the first AUG start codon, yielding a longer, 44 amino acid peptide (SprG1-long) and from an internal AUG start codon, producing a shorter, 31 residue peptide (SprG1-short) that is the more abundant form. The RNAs of this TA system were found to be constitutively expressed, with an approximately 12-fold shorter half-life of the SprF1 antitoxin transcript compared to the peptide encoding RNA. Inducible expression of SprG1 inhibited *S. aureus* growth, correlated with cell death. Specific duplex formation with SprF1 negatively regulates SprG1, presumably by RNA degradation. This is in contrast to the SprA/SprA1_AS_ system, in which inhibition of translation has been proposed to downregulate SprA1 [11,12,49]. Both SprG1 encoded peptides appear to be secreted pore forming toxins, with the longer version showing higher lysis activity against human erythrocytes. Together with an observed activity of the SprG1 peptides against Gram positive and Gram negative bacteria, this finding highlights that SprF1/SprG1 extends beyond a *sensu stricto* TA system, since SprG1 encoded peptides fulfill characteristics of virulence factors [49]. Triggers and functions of the SprF1/SprG1 module are yet to be identified.

## 3. Type II TA Systems

Currently three main groups of type II toxin-antitoxin systems have been experimentally verified in *S. aureus*, namely MazEF/PemIK, YefM-YoeB (AxeTxe) and Omega-Epsilon-Zeta. All groups possess proteic toxin and antitoxin components and are found in common *S. aureus* strains [22,50]. The MazEF/PemIK systems have been studied extensively, whereas considerably less is known about YefM-YoeB of *S. aureus*. The tripartite Omega/Epsilon/Zeta TA system has so far not been well experimentally characterized.

### 3.1. MazEF and PemIK

MazEF was originally described in *E. coli* and was the first chromosomal TA system, or “addiction module” reported [51]. In *S. aureus*, the toxin component *mazF* of the *mazEF* locus was first discovered and described as an ORF of unknown function during the inspection of the *sigB* locus that encodes the alternative sigma factor and stress regulator σ^B^ [52]. A later study revealed a relation between *mazF* and the *pemK* toxin gene from *E. coli* and identified another small ORF, the antitoxin *mazE*, upstream of *mazF* [53]. Based on these results, the prevalence of this TA loci in bacteria was shown shortly thereafter [54] and it seems likely that the *mazEF* locus is present in all Staphylococcal species [50,55].

**Figure 2:**
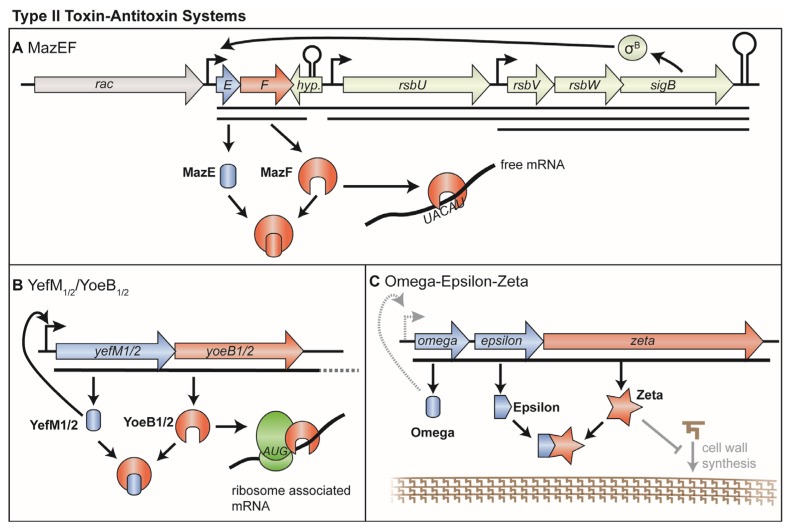
Type II toxin-antitoxin systems in *S. aureus* (**A**) The MazEF toxin-antitoxin system is embedded in the *rsbUVWsigB* locus. One promoter drives *mazEF* transcription, which can comprise the downstream *rsbUVWsigB* genes depending on a weak transcriptional terminator and transcriptional read-through. Free toxin MazF cleaves available mRNA at UACAU sites and can be inhibited by the antitoxin protein MazE. The system is negatively regulated by the *sigB* encoded σ^B^; (**B**) The toxin YoeB of the YefM-YoeB toxin-antitoxin system is a ribosome dependent RNase that cleaves close to the start codon. The antitoxin YefM inhibits the toxin by protein-protein interactions and can auto-regulate its own operon; (**C**) The Omega-Epsilon-Zeta system. In contrast to many other type II TA-operons, this is a tripartite system, where the regulation of the operon is separate from the antitoxin protein. The Omega protein is thought to auto-regulate its own operon, whereas the antitoxin Epsilon is supposed to inhibit toxicity from the Zeta toxin. This system has not been studied in depth, therefore most elements depicted here are based on predictions and homology to closely related systems. Unclear elements and functions are indicated by faint color. Systems are not drawn to scale.

Transcription of *mazEF* and the genes of the *sigB* locus (*rsbU*, *V*, *W* and *sigB*) is coupled, especially during heat shock [56,57]. The *mazEF-rsbUVW-sigB* operon (Figure 2A) is proposed to possess at least three promoters, one upstream of *mazEF*, one of *rsbU* and one of *rsbV*, as well as a Rho-independent transcriptional terminator downstream of *sigB* [57,58]. In addition, a weak transcriptional terminator is predicted downstream of the *mazEF* genes, permitting transcriptional read-through due to its low Gibbs free energy [57,59]. This results in several transcripts comprising either only *mazEF* or, *mazEF* and an increasing number of *sigB* related genes. Unexpectedly, the *mazEF* promoter is needed for full activity of the SigB system [57]. In addition, the *mazEF* promoter is not auto-regulated by its antitoxin MazE, as in many other TA systems, but instead inhibited by σ^B^. In contrast, both SarA, a transcriptional regulator and sub-MIC concentrations of erythromycin and tetracycline stimulate *mazEF* transcription [57].

Although transcription levels can serve as an indicator, activity of TA system toxin components are strongly dependent on the levels of toxin and antitoxin proteins in the cell. In type II TA systems, the unstable antitoxin is continuously degraded by a protease. Upon environmental stresses, replenishment is insufficient, which shifts toxin-antitoxin ratios and thus unleashes the toxin [5,6]. In *S. aureus*, MazE possesses a very short half-life of approximately 18 min, which is considerably shorter compared to its *E. coli* orthologue [60]. MazE levels stayed steady in a knockout strain of the ClpP protease and decreased only slowly in a knockout of the chaperone ClpC, indicating that this proteolytic complex aids in the degradation of MazE [60].

The recently solved structure of MazF from *S. aureus* revealed a high structural similarity to its *Bacillus subtilis* orthologue with a typical MazF/CcdB fold [61,62,63,64]. MazF was found to dimerize to a rigid complex by several inter-molecular contacts. Based upon the NMR and the structural data, *S. aureus* MazE antitoxin is thought to bind in the same manner to its MazF toxin as in the *Bacillus* orthologue.

Initial investigations on *S. aureus* MazF were conducted *in vitro* and in *E. coli*. A first study already demonstrated RNase activity [65] and later investigations indicated that UACAU is the preferred substrate of *S. aureus* MazF *in vivo* [55], although others sites were shown to be cleaved *in vitro* as well [66,67]. Overexpression of MazF in *S. aureus* has been reported to decrease the amount of CFU, while cells were reported to be alive in a live-dead staining for at least one hour [68]. It was therefore proposed that mRNA cleavage by MazF inhibits growth. An investigation of transcript levels in *S. aureus* revealed that *hla*, *spa* and *sigB* mRNA amounts were decreased, while levels of *sarA*, *recA* and *gyrB* remained steady upon induction of *mazF*. In accordance, the protein levels of SigB were decreased and those of SarA were increased over a time course of 90 minutes [68]. Direct cleavage of the *spa* and *rsbW* transcripts *in vivo* by MazF at UACAU could be demonstrated but some other identical pentad sequences remained uncut [55]. It has been proposed that one or more RNA binding proteins may serve to protect MazF cleavage sites [68] or secondary structure formation of RNAs may prevent access of the nuclease. Accordingly, a recent publication highlights the possibility that some RNAs might be protected by the helicase CshA and this may therefore shape the affected pool of RNAs [69]. Interestingly, cleavage of UACAU sites can be detected in a WT strain without overexpression of the toxin or deletion of the antitoxin [55] which suggests that MazF might play a regulatory role for certain transcripts even under non-stress conditions. Because of the abundance of UACAU sites in the pathogenicity factor *sraP* gene [66] and clustering in functional association networks [70], an involvement of *mazEF* in pathogenicity has been proposed. However, since the association of *mazEF* with *sigB* is highly conserved also in other *Staphylococcus* species and since *mazEF* is also functional in the non-pathogenic *S. equorum* [36], it remains to be worked out to what extent pathogenesis and cell physiology are regulated by *mazEF*.

The *pemIKsa* system from *S. aureus* CH91 is related to the canonical *mazEF* system, but differs significantly in its amino acid sequence. It was initially discovered on the plasmid pCH91, but is also present on other *S. aureus* plasmids and can be found in the chromosomes of some staphylococcal species [71]. As expected from a plasmid borne TA system, the *pemIKsa* locus is able to stabilize episomal elements as shown in *E. coli*. Overexpression of the toxin PemKsa leads to a growth defect in *S. aureus*, presumably caused by the RNase activity of PemKsa, which can be counteracted by its antitoxin PemIsa. PemKsa recognizes the target RNA by the sequence UAUU and cleavage of *scpB*, *adhC*, *ftsW* transcripts was demonstrated *in vitro*. The degradation of UAUU containing transcripts could be confirmed in *E. coli*, where *scpB* and *argR* mRNA levels were shown to decrease upon *pemKsa* expression. However, when cleavage capability was tested in *S. aureus*, the previously cleavable transcript *argR* was not degraded, whereas *scpB* and *ftsW* transcripts could still be cleaved. This suggests that PemKsa cleavage is differently regulated in *S. aureus*, similar to what was demonstrated for the *mazEF* system [55,68]. Interestingly, the *pemIsa* gene transcript is not susceptible to PemKsa, whereas the downstream part containing the *pemKsa* transcript is degraded. In addition, in *E. coli*, the antitoxin PemIsa can be produced even if induced three hours after the onset of toxin PemKsa expression. It was therefore proposed, that PemKsa production is auto-regulated by the cleavage of the *pemKsa* transcript. This would downregulate PemKsa synthesis while continuously producing the antitoxin PemIsa and therefore restore cell growth [71]. Although a link to pathogenicity has been hypothesized, it remains unclear what physiological role this system has as a plasmid encoded element in *S. aureus*.

### 3.2. YefM-YoeB (Axe-Txe)

The *yefM-yoeB* TA systems (also known as *axe/txe*) are widespread in bacteria and the presence in the *S. aureus* genome was first inferred by homology from a plasmid that encoded the orthologous *Francisella phd/yefM* TA system [72]. Shortly thereafter, an ortholog was discovered in *Enterococcus* which was named *axe/txe*, and the homology to the *S. aureus* system was also noted [73]. Owing to the independent descriptions of the *S. aureus* orthologs, the TA system is referred to as either YefM-YoeB or Axe-Txe in literature. To date two paralogous chromosomally encoded systems have been described, named *yefM-yoeB-sa1* (*axe-txe1*) and *yefM-yoeB-sa2* (*axe-txe2*) [22,57]. Both systems can be found at the same time in the same strains (Figure 2B).

The antitoxins YefM-sa1 and YefM-sa2 share 25% identity and 47% similarity with each other, while the toxin components YoeB-sa1 and YoeB-sa2 share 30% identity and 45% similarity [74]. The overexpression of both toxins leads to growth inhibition of *E. coli* that can be averted by simultaneously inducing the cognate antitoxin [74]. In contrast to some TA modules in *E. coli* [75] the two *S. aureus* YefM-YoeB paralogs do not cross-talk. *i.e.*, the antitoxin from one system cannot counteract the toxin from the other system and they also do not transcriptionally regulate each other. Instead both systems are transcriptionally auto-regulated by their cognate antitoxin [57,74].

It is still unclear, how the activities of the YefM-YoeB systems are modulated. Transcripts and antitoxin proteins of both systems are present under standard laboratory conditions, but in the presence of sub-MIC concentrations of the antibiotics erythromycin and tetracycline, their mRNA levels were increased [57]. Notably, the YefM-YoeB-sa2 system was detectable on two transcripts instead of only one. In addition to a smaller, expected transcript, a much longer RNA, comprising a gene annotated as endo-1,4-glucanase was detected, the abundance of which also increased upon antibiotic stress [57].

Regarding the stability of the antitoxins, it was observed that YefM-sa1 and YefM-sa2 possess half-lives that are much shorter compared to their *E. coli* counterparts and MazE [60]. This leaves room for speculation that these systems might be able to rapidly react to changing environmental conditions. The degradation of the antitoxin could be pinpointed to ClpP but in contrast to the *mazEF* system where ClpC supports degradation of MazE, ClpC is essential for YefM-sa1 and -sa2 proteolysis. At present it is unknown what triggers the degradation of the antitoxins to unleash the toxin activity of YoeB.

The YoeB protein from *E. coli* acts as a ribosome dependent RNase that inhibits growth and translation [76]. Expression of the *S. aureus* orthologs YoeB-sa1 and YoeB-sa2 in *E. coli* also leads to growth defects and cleavage close to the start codon, however other downstream sites are also targeted [74]. Despite a recently reported weak ribosome independent cleavage activity of YoeB-sa1 *in vitro*, the *S. aureus* orthologues probably exert their functions indeed by cleaving in complex with the ribosome [77]. Due to the nature of such cleavage it is reasonable to assume, that a large set of translationally active RNAs are controlled by YoeB. How this supposedly massive shutdown of translation relates to *S. aureus* physiology is yet to be determined. In comparison, the RNase MazF appears to be much more selective due to the recognition of a certain sequence.

### 3.3. Omega/Epsilon/Zeta

Omega/Epsilon/Zeta constitutes a three component TA system and therefore possesses an unusual organization (Figure 2C). As in most other systems, the toxin component, here called Zeta, is inhibited by the antitoxin component Epsilon. The third protein Omega, mediates regulation of the operon, which is in most other systems achieved by a bifunctional antitoxin, instead of an additional protein. To our knowledge, there has only been one described instance of the Omega/Epsilon/Zeta system in *S. aureus* [78]. The methicillin resistant strain CM05 possesses a chromosomally integrated broad host range plasmid pSM19035 from Streptococcus that contains the Omega/Epsilon/Zeta toxin-antitoxin system [78,79] by which this genetically unstable region might be maintained. Functional studies of the Zeta toxin that were done on the related pneumococcal PezAT system, revealed that the toxin phosphorylates the peptidoglycan precursor uridine diphosphate-N-acetylglucosamine and therefore inhibits peptidoglycan synthesis leading to autolysis [17]. A detailed assessment of how this TA system functions in *S. aureus* and its role is however still to be made.

## 4. Type III TA Candidate Systems

**Figure 3:**
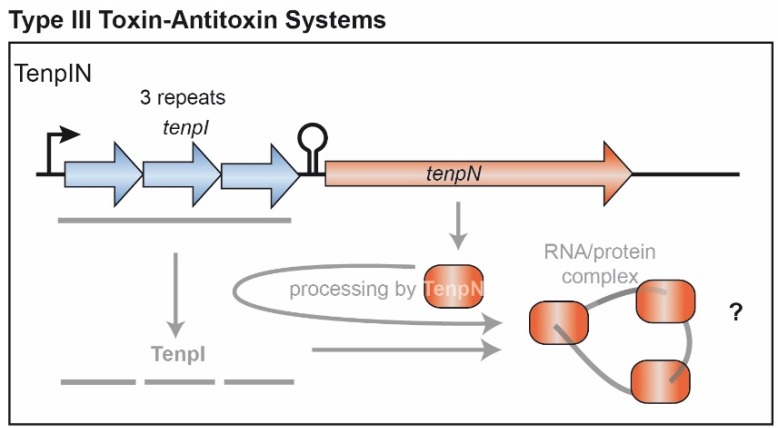
Model of the type III toxin-antitoxin system TenpIN in *S. aureus*. The antitoxin TenpI is predicted to possess three repeats that are proposed to be processed by the toxin TenpN. Presumably the processed TenpI RNA fragments are able to bind the toxin protein forming an RNA/protein complex. Note that this system has not been tested experimentally in *S. aureus* and the figure presented here is solely a model based on the predicted chromosomal regions and orthologous systems. Not drawn to scale.

In Type III TA systems, antitoxins are encoded by short tandem repeats upstream of the toxin gene [21]. These antitoxins form pseudoknots that inactivate the cognate toxin via RNA-protein interaction (Figure 3). The first TA system of this kind, ToxIN, was identified on a plasmid of the Gram negative plant pathogen *Pectobacterium atrosepticum* (formerly known as *Erwinia carotovora*) [80]. The ToxN toxin has endonucleolytic activity and plays a critical role in the induction of cell death upon phage infection. An *in silico* study discovered more than 100 type III TA systems throughout the bacterial domain [21]. According to their primary structures, a total of three type III TA system families have been categorized and functionally analyzed in *E. coli*. Representatives of the *tenpIN* family have also been detected in *S. aureus* genomes.

The eponymous member of the *tenpIN* family was discovered in *Photorhabdus luminescens* and termed according to its function as Type III ENdogenous to Photorhabdus Inhibitor/toxiN. *tenpIN* loci were identified in three different *S. aureus* strains, located on plasmids or in the chromosome [21]. The plasmid pPR9 borne *tenpN* gene encodes a putative protein of 249 amino acids in size, whereas a 259 residue TenpN homolog may be expressed from the chromosome of *S. aureus* strain A9754 and from plasmids including VRSAp, pSK41, pGO1. Studies on type III TA systems in other Gram positive bacteria have demonstrated anti-phage activity in *Lactococcus* [81] and functions in plasmid retention during sporulation of *Bacillus* *thuringiensis* [82]. However, these were due to other type III TA systems, but not members from the *tenpIN* family. So far no biochemical investigations of the proposed type III loci of staphylococci have been published and it will be interesting to learn, if they play a functional role.

## 5. Final Remarks

To date, six types of TA systems have been proposed and representatives of three of them have been identified in genomes of *S. aureus*. Regarding the abundance of these systems, there is considerable strain variation, which may resemble the lifestyles of *S. aureus* isolates stemming from different environments [22]. Although the TA systems of *S. aureus* are relatively well conserved, clear cut functions or phenotypes have so far been identified only parsimoniously. Several mutant strains with inactivated TA systems were generated and investigated, particularly pertaining to type II TA systems which were the first to be characterized in *S. aureus*. For example, a *mazEF* deletion strain was found to be decreased in β-lactam tolerance [44]. It has also been suggested that TA systems play a role in persister cell formation in *S. aureus* [68,83,84,85]. However, in contrast to *E. coli*, in which the consecutive deletion of type II TA systems encoding nucleases had resulted in a decrease of drug tolerant persister levels [86], a tripe knockout *S. aureus* strain, lacking *mazEF* and both *yefM-yoeB* paralogs did not exhibit this phenotype [87]. This may be due to another kind of persister mechanism in *S. aureus*, but it could also reflect that more TA systems remain hidden in the *S. aureus* genome, yet to be identified.

Following the notion that TA systems could be exploited for biotechnological purposes or as targets for novel anti-infectives [88,89,90,91], a number of approaches have also been taken in *S. aureus*. Bacconi *et al.* have cloned an *epsilon/zeta* containing TA system of an *E. faecalis* plasmid into an *S. aureus* vector for enhanced stability during murine infection [92]. Making use of an *S. aureus* encoded type I TA toxin, PepA1 (see above) served as a basis for the successful development of a cyclic pseudopeptide antibiotic, which is active against Gram positive and Gram negative bacteria [93]. The peptide was optimized to yield a compound with low hemolytic activity and enhanced stability in human serum, while retaining antimicrobial efficacy.

Future research on staphylococcal TA systems will need to address a number of major questions: What are the functions of the different staphylococcal TA systems? Are one or more of them just remnants of plasmid addiction systems, recalcitrant to evolutionary eradication [29], or are they associated with so far unassigned phenotypes? Are additional staphylococcal TA candidate loci proposed by *in silico* analysis functional? And if so, under which conditions and with which outcome? Are there more TA systems hidden in the genomes in *S. aureus* and other facultatively pathogenic staphylococci? Particularly TA systems with uncommon types [18,19,20] or possibly entirely novel modes of activity control may have gone unnoticed to date.

In the light of an unprecedented need for new antimicrobial compounds [94], TA systems are considered as an emerging source of both target structures and lead compounds. Future studies will hopefully shed more light on their functions in *S. aureus*.

## Abbreviations

The following abbreviations are used in this manuscript:

TA	toxin-antitoxin
